# Isotopic Evidence Suggests a High Contribution of Hypohalous Acids to Sulfate Formation in the Coastal Marine Boundary Layer

**DOI:** 10.1021/acs.est.5c14392

**Published:** 2026-04-02

**Authors:** Qianjie Chen, Allison R. Moon, Becky Alexander, Andrew Schauer, Men Xia, Yifan Jiang, Zhouxing Zou, Tao Wang

**Affiliations:** † Department of Civil and Environmental Engineering, 26680 The Hong Kong Polytechnic University, Hong Kong SAR 999077, China; ‡ Department of Atmospheric and Climate Science, 7284 University of Washington, Seattle, Washington 98195, United States; § Department of Earth and Space Sciences, University of Washington, Seattle, Washington 98195, United States; ∥ Nanjing-Helsinki Institute in Atmospheric and Earth System Sciences, 12581 Nanjing University, Suzhou 215000, China

**Keywords:** sulfate aerosol, hypohalous acid, oxygen isotope, marine boundary layer, reactive halogen

## Abstract

A potential impact of reactive halogens on climate and air quality is through oxidation of sulfur dioxide by hypohalous acids (HOX with X = Cl, Br, or I) to produce sulfate aerosols. This sulfate formation mechanism is typically not included in climate and air quality models, largely due to the limited observational evidence supporting its significance. In this study, we measured the oxygen isotopic composition of sulfate in aerosol particles as well as the concentrations of hypohalous acids during a field campaign in coastal Hong Kong. The oxygen isotopic signatures suggest that hypohalous acids account for more sulfate production than the traditional oxidant hydrogen peroxide (H_2_O_2_) in both clean air masses from the South China Sea and polluted air masses from the southeast coast of China. Throughout the campaign, the contribution of hypohalous acids to sulfate production is estimated to range from 30 to 76%. Among the hypohalous acids, the contribution of HOI to sulfate production is largest in clean air masses, whereas the contribution of HOI and HOCl are comparable in polluted air masses. Our results highlight the need for future measurements of HOI in the coastal marine boundary layer.

## Graphical abstract

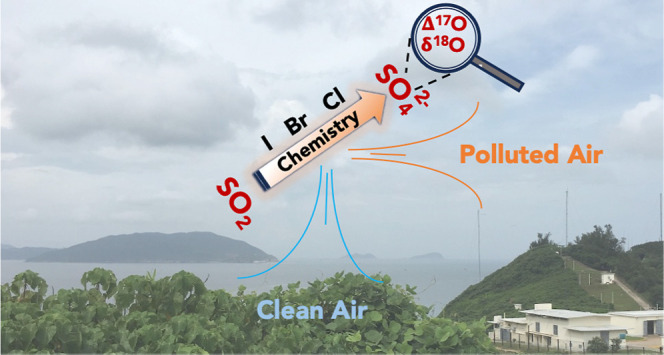

## 1 Introduction

Sulfate plays a key role in the formation, growth, and chemical properties of aerosol particles and cloud droplets in the troposphere, with profound implications for climate and air quality. Globally, the majority of sulfate is produced from the oxidation of sulfur dioxide (SO_2_) that is emitted from fossil fuel combustion and volcanoes or through oxidation of dimethyl sulfide (DMS) released from the marine biosphere.^1,2^ SO_2_ can be oxidized by a variety of oxidants to form sulfate in the atmosphere. In the gas phase, SO_2_ is oxidized by OH radicals to form sulfuric acid (H_2_SO_4_).^3^ In the presence of clouds, the dissolved SO_2_ (S­(IV) = SO_2_·H_2_O + HSO_3_^–^ + SO_3_^2–^) is oxidized in cloud droplets by hydrogen peroxide (H_2_O_2_), ozone (O_3_), hypohalous acids (HOX with X = Cl, Br, or I), and transition metal ion (TMI)-catalyzed O_2_.^4–6^ In the marine boundary layer (MBL), heterogeneous oxidation of SO_2_ by O_3_ can also produce sulfate on alkaline sea salt aerosols.^7,8^ Other important sulfate formation pathways in specific environments include (i) gas-phase oxidation of SO_2_ by Criegee intermediates^9^ and aqueous oxidation of S­(IV) by isoprene hydroxyl hydroperoxides and hydroxymethyl hydroperoxide^10,11^ over forested regions, and (ii) heterogeneous oxidation of SO_2_ by nitrogen dioxide (NO_2_) on alkaline aerosols,^12,13^ photosensitized oxidation of S­(IV) by humic-like substances on alkaline aerosols,^14^ uncatalyzed sulfite oxidation on alkaline aerosols,^15^ and interfacial SO_2_ oxidation involving O_2_ on acidic microdroplets under polluted haze conditions.^16^ After decades of research, the relative contributions of various sulfate formation pathways remain unclear.

About three decades ago, Vogt et al.^54^ proposed that HOBr and HOCl could efficiently oxidize SO_2_ on sea salt aerosols and cause a reduction of cloud condensation nuclei in the remote MBL. The follow-up one-dimensional modeling study suggested HOBr and HOCl accounts for ∼30% of the sulfate production in both cloudy and cloud-free MBL.^50^ In a global chemical transport model, the fraction of sulfate produced from HOBr oxidation could reach up to 45% in the subtropical MBL.^55^ More recently, a box modeling study suggested HOI contributes 57% of sulfate production in the mixed urban and maritime coastal environment.^6^ The observational constraint on the HOX contribution to sulfate production was provided by Chen et al.^5^ based on their observations of Δ^17^O_nssSO4_, which suggested 30–50% of sulfate in the remote MBL was produced via the HOX oxidation mechanism. The climate implication of sulfate production via the HOX oxidation mechanism has not been assessed.

The oxygen isotopic composition of sulfate can be used to constrain sulfate formation mechanisms because the oxidants contributing to the oxidation of SO_2_ to sulfate have unique oxygen isotopic signatures.^5,7,17–19^ Throughout the manuscript, we use δ^18^O, δ^17^O, and Δ^17^O notation to represent oxygen isotopic signatures of sulfate

δxO=(Ox/O16)Sample(Ox/O16)VSMOW E1

E2 $$\Delta^{17}O=\delta^{17}O-0.52\delta^{18}O$$

where *x* = 17 or 18 and VSMOW stands for Vienna Standard Mean Ocean Water. In the gas phase, SO_2(g)_ exchanges its oxygen isotopes rapidly with water vapor (H_2_O_(g)_) so that δ^18^O and Δ^17^O of SO_2(g)_ depend on those of H_2_O_(g)_.^19^ The oxygen isotopic signatures of H_2_SO_4(g)_ formed from the OH oxidation pathway thus depend strongly on those of H_2_O_(g)_ since OH also exchanges its oxygen atom with H_2_O_(g)_.^3,17,20^ In the aqueous phase, S­(IV) exchanges its oxygen isotopes rapidly with liquid water (H_2_O_(l)_) so that δ^18^O and Δ^17^O of S­(IV) depend on those of H_2_O_(l)_.^18,21^ During S­(IV) oxidation, the oxygen atoms from the oxidant could be transferred to the product sulfate. In particular,H_2_O_2_, O_3_, and TMI-catalyzed O_2_ will transfer two, one, and one oxygen atom to sulfate, respectively.^17,18^ HOX promotes sulfate formation via nucleophilic attack of HSO_3_^–^/SO_3_^2–^ onto the X (X = Cl, Br, or I) atom followed by rapid hydrolysis of XSO_3_^–^,^22–24^ and thus will not transfer its oxygen atom to sulfate. The oxygen isotopes offer a key discriminating feature for determining the relative importance of sulfate formation pathways. The oxygen isotopic signatures of sulfate from different pathways are shown in Table S1. Once formed, sulfate does not undergo oxygen isotopic exchange, including exchange with water in clouds and aerosols.^69^

In China, more than 300 million people are living within 100 km from the coast.^25^ Pearl River Delta–Hong Kong (PRD-HK) is a highly populated and urbanized coastal region in Southern China. Despite the implementation of emission control policies, fine particulate matter (PM_2.5_) concentrations in PRD-HK (20–30 μg m^–3^ annual mean with ∼30% as sulfate) are still much higher than the World Health Organization guideline (5 μg m^–3^).^26,27^ Understanding how sulfate is produced will improve PM_2.5_ prediction under different emission control scenarios in the PRD-HK region, and possibly other coastal cities in China and the world. Previous field studies reported high levels of reactive halogens in coastal Hong Kong, as a consequence of the interactions between anthropogenic nitrogen oxides (NO_*x*_) and natural sea salt.^28–31^ HOX is a main reservoir of reactive halogens in the MBL.^28^ It was calculated in a box modeling study that HOX dominates sulfate production in the polluted MBL,^6^ but observational evidence is lacking. In this study, we measured the oxygen isotopes (^16^O, ^17^O, ^18^O) of sulfate in PM_2.5_ as well as HOCl and HOBr at a coastal site in Hok Tsui, Hong Kong during August–November 2021. This study provides an observational constraint on the contribution of HOX to sulfate formation mechanisms in the coastal MBL of Southern China.

## 2 Method

### 2.1 Aerosol Sampling in Hong Kong

PM_2.5_ (particulate matter with diameter <2.5 μm) was sampled daily at the Hok Tsui Supersite (22.21 °N, 114.25 °E) in coastal Hong Kong during three selected periods in 2021: (I) August 13–24, (II) September 28–October 8, and (III) October 28–November 1. A Partisol 2025i-D Dichotomous Sequential Air Sampler (Thermo Scientific Inc.) was used to collect 24 h integrated samples (00:00–23:59) on Teflon filters (Pall Corporation, Michigan). The site is about 200 m away from the coast, surrounded by ocean water, vegetation, and sparse country roads. The nearest urban center is about 15 km to the north while the highly urbanized Pearl River Delta region is within 200 km to the north. Air masses arriving at the site were relatively clean ([PM_2.5_] = 5.7 ± 0.8 μg m^–3^, [SO_4_^2–^] = 1.5 ± 0.3 μg m^–3^) from the South China Sea during Period I and more polluted ([PM_2.5_] = 21.8 ± 5.8 μg m^–3^, [SO_4_^2–^] = 5.9 ± 1.5 μg m^–3^) from the southeast coast of China during Periods II and III (Figure 1). The average air temperature and relative humidity during the sample collection period were 28 ± 3 °C and 84 ± 8%, respectively.

**Figure 1:**
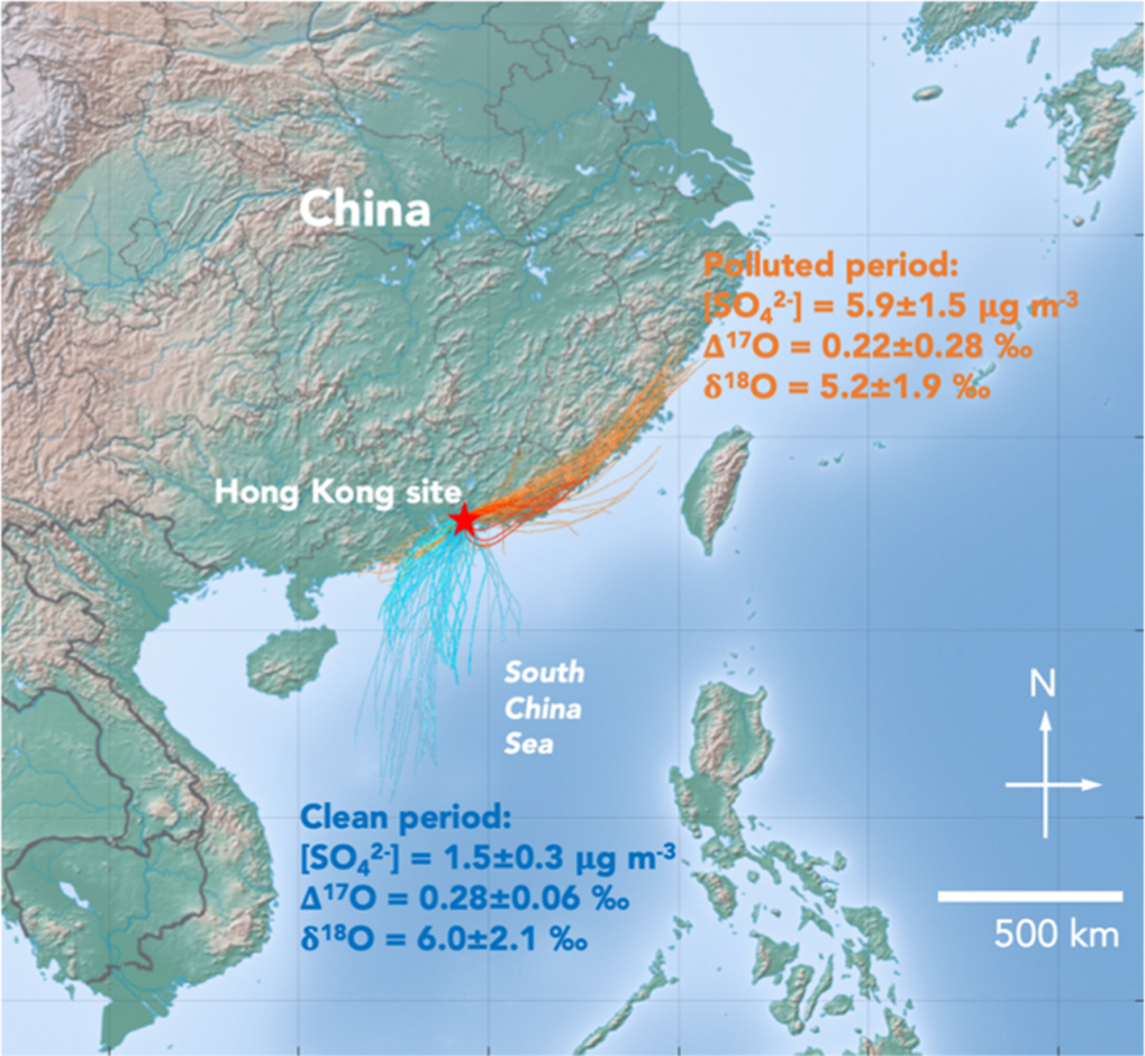
Location of the Hong Kong field site (red star) and the 24 h HYSPLIT air parcel back trajectories during the aerosol sampling period. Trajectories for the clean Period I (August 13–24) are shown in cyan, while those for polluted Period II (September 28–October 8) and III (October 28–November 1) are shown in orange. The periods August 21–24 and September 28 are highlighted in deep cyan and deep brown, respectively.

### 2.2 HOCl and HOBr Measurements

An iodide-adduct ToF-CIMS was deployed at the Hok Tsui supersite during the aerosol sampling period to measure reactive halogen species including HOCl and HOBr, as described in Xia et al.^31^ Air was sampled at 1.5 m above the ground through a 0.5 m long, 1.1 cm inner diameter PFA tube at 25 L min^–1^, of which 2 L min^–1^ was sampled into the ToF-CIMS where IHOCl^–^ and IHOBr^–^ clusters were detected. The sampling tube was replaced every 2 days to reduce inlet artifact influence from deposited particles, given the practical constraints of the field campaign.^30,31^ The sampling losses of HOCl and HOBr, determined by injecting HOCl and HOBr reference gases into the used sampling inlet,^30,31^ were 13% and 30%, respectively. The instrument background signals of HOCl and HOBr were determined by injecting zero air to the ToF-CIMS every 2 days. The IHO^37^Cl^–^ signals correlate well with IHO^35^Cl^–^ signals with a slope of 0.33 (Figure S3), very similar to the theoretical ratio (0.33). For calibration purposes, HOCl was generated by bubbling N_2_ gas through NaOCl solution (6 mM buffered to pH = 6.8) while HOBr was generated by bubbling N_2_ gas through silver nitrate solution mixed with liquid Br_2_.^30,31^ The 3σ limit of detection (LOD) was 1.4 ppt for HOCl and 1.9 ppt for HOBr, respectively. The time series of HOCl mixing ratio during the study period is shown in Figure S2. The HOBr signals were very low due to low abundance so that the interference from other species could be large. Therefore, we only report the upper limits of HOBr mixing ratio in this study: 0.5 ± 0.5 ppt in clean air masses and 0.7 ± 0.7 ppt in polluted air masses.

### 2.3 Laboratory Measurements of Oxygen Isotopes of Sulfate

The oxygen isotopes of sulfate were measured at University of Washington IsoLab, following our previous protocols.^5,32,33^ Briefly, sulfate on the filters was dissolved in 18 MΩ water and organics were removed by adding 30%H_2_O_2_ after conversion into stable sodium sulfate through cation exchange resin. The sample was then purified using a Dionex ICS-2000 ion chromatograph, converted to Ag_2_SO_4_ using a cation exchange resin, and then dried to solid in quartz capsules for oxygen isotopic measurements. Due to low sulfate abundance, samples of consecutive days August 13–17, August 18–20, August 21–24, and October 03–04 were grouped into four separate sets in order to have enough sulfate for isotopic analysis. The remaining eight samples each contained sufficient sulfate for isotopic analysis. Period I includes three samples (Aug. 13–17, Aug. 18–20, Aug. 21–24); Period II includes five samples (Sept. 28, Sept. 30, Oct. 1, Oct. 2, Oct. 3–4); and Period III includes four samples (Oct. 28, Oct. 29, Oct. 31, Nov. 1). Individual quartz capsules containing solid Ag_2_SO_4_ samples were dropped into a Temperature Conversion/Elemental Analyzer (TC/EA) where Ag_2_SO_4_ was pyrolyzed at 1000 °C to form Ag_(s)_ + SO_2(g)_ + O_2(g)_. The O_2(g)_ was carried by the continuous helium flow to a Finnigan MAT 253 stable isotope ratio mass spectrometer (IRMS) for determination of ^16^O, ^17^O, and ^18^O abundances, from which δ^18^O and Δ^17^O were calculated. We used the IAEA-N-1 stable isotope reference material for δ^18^O calibration and three interlaboratory calibrated Δ^17^O standards (Sulf-α, Sulf-β, and Sulf-ε) for Δ^17^O calibration (Table S2).^32,33^ The effects of oxygen isotope exchange in quartz capsules during pyrolysis on δ^18^O and Δ^17^O were considered, using 11 reference materials measured in both quartz and gold capsules (Table S2), following previous work.^33,34^ The uncertainties of δ^18^O and Δ^17^O, are ±1.4‰ and ±0.3‰, respectively, considering replicate analyses of standards and isotopic exchange in quartz capsules.

The nonsea-salt sulfate δ^18^O_nssSO4_ and Δ^17^O_nssSO4_ were calculated by subtracting the sea-salt sulfate fraction with δ^18^O_ssSO4_ = 9‰ and Δ^17^O_ssSO4_ = 0‰.^35^ The sea-salt sulfate fraction, calculated using the mass ratios of [ssSO_4_^2–^]/[Na^+^] = 0.252 and [ssSO_4_^2–^]/[Mg^2+^] = 2.115 in seawater,^36^ is only 4 ± 5% on average during the study period. The [SO_4_^2–^], [Na^+^], and [Mg^2+^] concentrations were measured hourly with an online MARGA ion chromatography system (Metrohm Applikon B·V., The Netherlands). The aerosol pH was calculated using the E-AIM aerosol thermodynamic model (Model IV).^59^

### 2.4 Isotopic Calculations

The δ^18^O of rainwater (H_2_O_(l)_) collected at Hong Kong King’s Park located ∼20 km north from our site was measured and provided by International Atomic Energy Agency (IAEA) to be −6‰ in August and −10‰ in October 2021 (https://websso.iaea.org/). Here we assume δ^18^O_H2O(l)_ = −6‰ in Period I and −10‰ in Periods II and III. From isotopic equilibrium between H_2_O_(g)_ and H_2_O_(l)_, we calculate δ^18^O_H2O(g)_ = −15‰ in Period I and −19‰ in Periods II and III (*E*3).^37^ The δ^18^O of SO_2(g)_ and S­(IV), which are in isotopic equilibrium with water vapor and cloudwater, can then be calculated in *E*4 and *E*5 (Figure S4).^18,19^

E3 $$\delta^{18}O_{H2O\left(g\right)}=\frac{1+{\;\delta }^{18}O_{H2O\left(l\right)}}{e^{\left[-7.685+6.7123\times \left(\frac{10^{3}}{T}\right)-1.6664\times \left(\frac{10^{6}}{T^{2}}\right)+0.35041\times \left(\frac{10^{9}}{T^{3}}\right)\right]\times 10^{-3}}}-1$$

E4 $$\delta^{18}O_{\text{SO}2g}=0.94\delta^{18}O_{H2O\left(g\right)}+24.2$$

E5 $$\delta^{18}O_{S\left(IV\right)}=0.95\delta^{18}O_{H2O\left(l\right)}+7.78$$

From previous experimental results, the δ^18^O of H_2_SO_4_ produced from SO_2(g)_ oxidation by OH (δ^18^O_H2SO4_) increases linearly with δ^18^O_H2O(g)_ (*E*6).^19^ The δ^18^O of sulfate produced from H_2_O_2_ oxidation (δ^18^O_SO4-H2O2_) in cloud droplets acquires 1/2 of the H_2_O_2_ signature^38^ (δ^18^O_H2O2_ = 35 ± 8‰) and 1/2 of the S­(IV) signature,^17^ as calculated in *E*7. The δ^18^O of sulfate produced from O_3_ oxidation (δ^18^O_SO4-O3_) or TMI-catalyzed O_2_ oxidation in cloud droplets acquires 1/4 of the oxidant signature (δ^18^O_O3_ = 128 ± 12‰; δ^18^O_O2_ = 24.6‰)^39–41^ and 3/4 of S­(IV) signature,^17^ as calculated in *E*8 and *E*9. Since HOX promotes sulfate formation via nucleophilic attack instead of transferring its own oxygen atom, the δ^18^O of sulfate produced from HOX oxidation (δ^18^O_SO4‑HOX_) in cloud droplets acquires 1/4 of δ^18^O_H2O(l)_ signature and 3/4 of S­(IV) signature,^22–24^ as calculated in *E*10. Evaporation of cloudwater will not change the oxygen isotopic signature of sulfate. Equilibrium fractionation between condensed-phase sulfate (in cloud droplets or aerosols) and gas-phase H_2_SO_4_ can be neglected because the gas-phase fraction is negligible. Primary sulfate from fossil fuel combustion at high temperature is expected to exhibit the O_2_ signatures (Δ^17^O = 0‰; δ^18^O = 23.9‰).^8,33,40^ Note that Sun et al.^63^ recently measured δ^18^O of sulfate from soot samples collected from the inner walls of chimneys in China and found a low δ^18^O value of about 13‰. However, the sulfate present on these soot samples may not accurately represent primary sulfate emitted from the chimney flue gas^64,65^ as sulfate can continue to form on soot deposited on the inner walls of the chimney.^64–66^ The δ^18^O of sulfate produced from different pathways are shown in Table S1.

E6 $$\delta^{18}O_{H2\text{SO}4}=0.71\delta^{18}O_{H2O\left(g\right)}+16.5$$

E7 $$\delta^{18}O_{\text{SO}4-H2O2}=0.5\delta^{18}O_{H2O2}+0.5\delta^{18}O_{S\left(IV\right)}$$

E8 $$\delta^{18}O_{\text{SO}4-O3}=0.25\delta^{18}O_{O3}+0.75\delta^{18}O_{S\left(IV\right)}$$

E9 $$\delta^{18}O_{\text{SO}4-TMI}=0.25\delta^{18}O_{O2}+0.75\delta^{18}O_{S\left(IV\right)}$$

E10 $$\delta^{18}O_{\text{SO}4-HOX}=0.25\delta^{18}O_{H2O\left(l\right)}+0.75\delta^{18}O_{S\left(IV\right)}$$

The δ^18^O_nssSO4_ is the overall signature of sulfate formed via different pathways (*E*11). Similar relationships apply to Δ^17^O_nssSO4_ (*E*12). The Δ^17^O of sulfate produced from H_2_O_2_ and O_3_ oxidation is 0.7‰ and 9.8‰, respectively (Table S1).^17,39^ Sulfate from other sources, including primary sulfate emitted from fossil fuel combustion and secondary sulfate produced from OH, TMI-catalyzed O_2_, and HOX oxidation mechanisms possess Δ^17^O of 0‰ (Table S1).^17^

E11 $$\delta^{18}O_{nssSO4}=f_{primary}\delta^{18}O_{primary}+f_{OH}\delta^{18}O_{H2\text{SO}4}+f_{H2O2}\delta^{18}O_{\text{SO}4-H2O2}+f_{O3}\delta^{18}O_{\text{SO}4-O3}+f_{TMI}\delta^{18}O_{\text{SO}4-TMI}+f_{HOX}\delta^{18}O_{\text{SO}4-HOX}$$

E12 $$\Delta^{17}O_{nssSO4}=f_{H2O2}\Delta^{17}O_{\text{SO}4-H2O2}+f_{O3}\Delta^{17}O_{\text{SO}4-O3}$$

E13 $$f_{primary}+f_{OH}+f_{H2O2}+f_{O3}+f_{TMI}+f_{HOX}=1$$

where *f*_primary_, *f*_OH_, *f*_H2O2_, *f*_O3_, *f*_TMI_, and *f*_HOX_ refer to the fraction of sulfate from primary sulfate and OH, H_2_O_2_, O_3_, TMI-catalyzed O_2_, and HOX oxidation mechanisms, respectively.

Since HOX oxidation produces sulfate with lowest δ^18^O, *f*_H2O2_ reaches maximum (*f*_H2O2,max_) by assuming *f*_primary_, *f*_OH_, and *f*_TMI_ to be zero and solving *E*11–*E*13. For each *f*_H2O2_ solution between zero and *f*_H2O2,max_, we can calculate *f*_O3_ from *E*12 and the *f*_HOX_ solution range (*E*14–*E*15). Substituting *f*_primary_ from *E*13 into *E*11 yields *E*14. Because (δ^18^O_SO4‑TMI_–δ^18^O_primary_) and (δ^18^O_H2SO4_–δ^18^O_primary_) are both negative, *f*_HOX_ reaches maximum when *f*_TMI_ and *f*_OH_ are zero. Similarly, substituting *f*_OH_ from *E*13 into *E*11 yields *E*15. Because (δ^18^O_SO4‑TMI_–δ^18^O_H2SO4_) and (δ^18^O_primary_–δ^18^O_H2SO4_) are both positive, *f*_HOX_ reaches minimum when *f*_TMI_ and *f*_primary_ are zero. For each sample, the median *f*_H2O2_, *f*_HOX_, and *f*_Other_ (=1–*f*_H2O2_–*f*_HOX_) values and uncertainties (expressed as the half-widths of the 95% confidence intervals) after propagating analytical uncertainties (Section 2.3) of δ^18^O_nssSO4_ (1.4‰) and Δ^17^O_nssSO4_ (0.3‰) are shown in Table S6.

E14 $$f_{HOX}=\left[\left(\delta^{18}O_{\text{SO}4-TMI}-\delta^{18}O_{primary}\right)f_{TMI}+\left(\delta^{18}O_{H2SO4}-\delta^{18}O_{primary}\right)f_{OH}+\left(\delta^{18}O_{\text{SO}4-H2O2}-\delta^{18}O_{primary}\right)f_{H2O2}+\left(\delta^{18}O_{\text{SO}4-O3}-\delta^{18}O_{primary}\right)f_{O3}+\left(\delta^{18}O_{primary}-\delta^{18}O_{nssSO4}\right)\right]/\left(\delta^{18}O_{primary}-\delta^{18}O_{HOX}\right)$$

E15 $$f_{HOX}=\left[\left(\delta^{18}O_{\text{SO}4-TMI}-\delta^{18}O_{H2\text{SO}4}\right)f_{TMI}+\left(\delta^{18}O_{primary}-\delta^{18}O_{H2\text{SO}4}\right)f_{primary}+\left(\delta^{18}O_{\text{SO}4-H2O2}-\delta^{18}O_{H2\text{SO}4}\right)f_{H2O2}+\left(\delta^{18}O_{\text{SO}4-O3}-\delta^{18}O_{H2\text{SO}4}\right)f_{O3}+\left(\delta^{18}O_{H2SO4}-\delta^{18}O_{nssSO4}\right)\right]/\left(\delta^{18}O_{H2SO4}-\delta^{18}O_{HOX}\right)$$

## 3 Results and Discussion

### 3.1 Oxygen Isotopic Signatures of Sulfate

The Hok Tsui field site was influenced by both clean air masses from the South China Sea ([SO_4_^2–^] = 1.5 ± 0.3 μg m^–3^) and polluted air masses from the southeast coast of China ([SO_4_^2–^] = 5.9 ± 1.5 μg m^–3^) during the study period (Figure 1). The Δ^17^O of nonsea-salt sulfate (Δ^17^O_nssSO4_) ranges from −0.35 to 0.66‰, with an average of 0.24 ± 0.26‰, for the PM_2.5_ samples collected at the site (Figure 2). Δ^17^O_nssSO4_ is not statistically different within clean and polluted air masses. Although such low Δ^17^O_nssSO4_ has been occasionally observed in the remote MBL,^5,42^ it is at the lower end of Δ^17^O_nssSO4_ observed in various environments around the globe so far.^43^ In comparison, Δ^17^O_nssSO4_ of 1.2 ± 0.1‰, 0.9 ± 0.3‰, and 0.9 ± 0.2‰ were measured in East China,^44^ North China,^45^ and coastal California,^8^ respectively.

**Figure 2:**
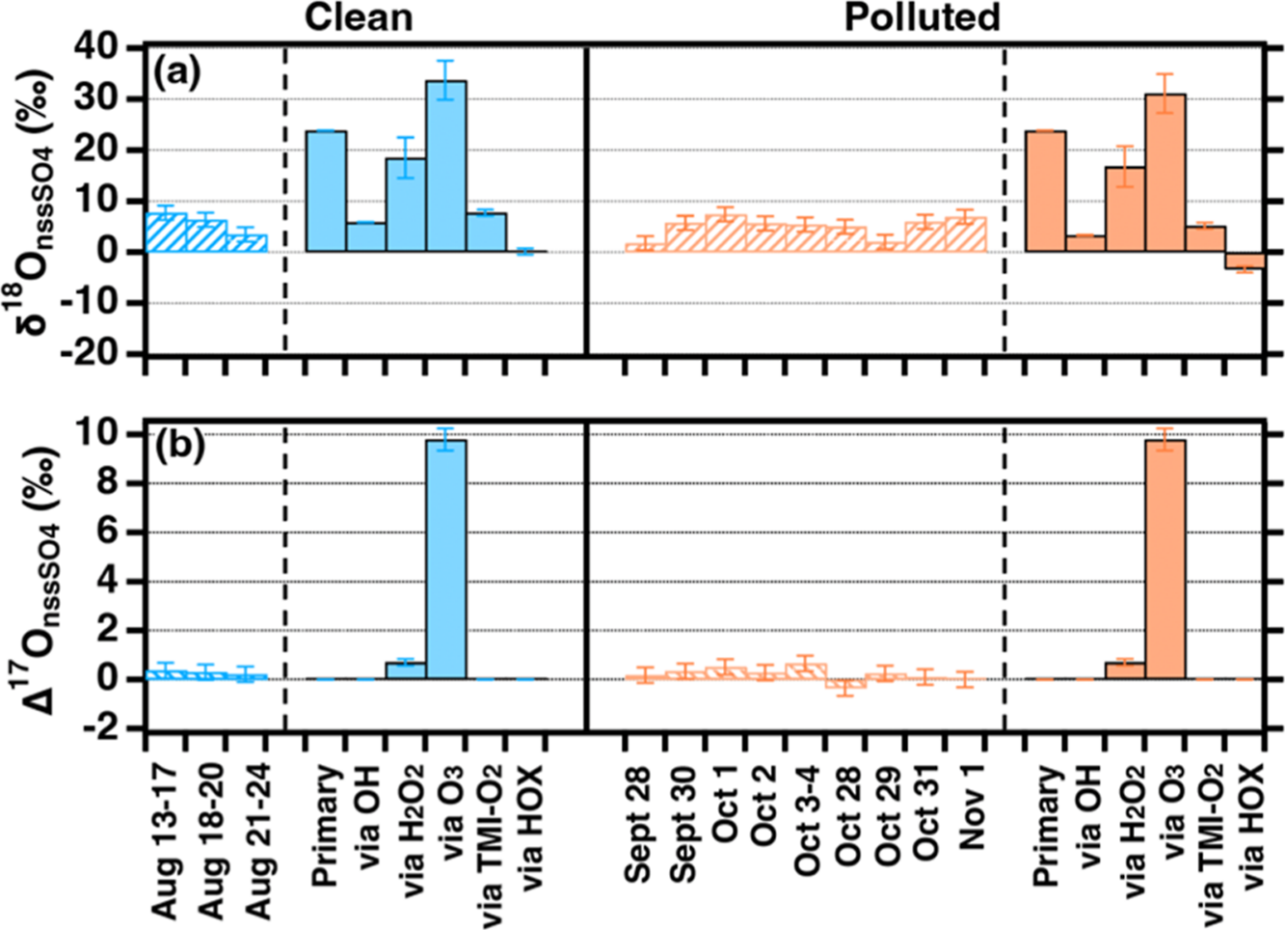
Observed δ^18^O_nssSO4_ and Δ^17^O_nssSO4_ for PM_2.5_ samples collected in Hong Kong, along with theoretical values for sulfate from different pathways during clean period and polluted period, respectively.

The δ^18^O of nonsea-salt sulfate (δ^18^O_nssSO4_) ranges from 2.0 to 7.7‰, with an average of 5.3 ± 2.0‰ (Figure 2), also showing no statistical difference within clean and polluted air masses. δ^18^O_nssSO4_ is substantially lower than observations in East China (10.0 ± 2.6‰),^44^ North China (11.1 ± 2.4‰),^46^ and coastal California (12.9 ± 1.9‰).^8^ The δ^18^O_nssSO4_ is affected not only by sulfate formation pathways, but also the δ^18^O of cloudwater and water vapor in the atmosphere, which has strong latitudinal and regional variations.^47^ Since δ^18^O of rainwater has been measured in this study, the δ^18^O_nssSO4_ and Δ^17^O_nssSO4_ can be combined to gain deeper insight into sulfate formation mechanisms (*E*11–*E*13). For each sample, we calculated the fraction of sulfate produced from H_2_O_2_ oxidation (*f*_H2O2_), HOX oxidation (*f*_HOX_), and other pathways (*f*_Other_ = *f*_primary_ + *f*_OH_ + *f*_TMI_ + *f*_O3_) by solving *E*11–*E*13. Explicit solutions for *f*_H2O2_, *f*_HOX_, *f*_primary_, *f*_OH_, *f*_TMI_, and *f*_O3_ are not possible since there are 6 unknows but only 3 equations. Instead, we calculate the possible ranges of *f*_H2O2_, *f*_HOX_, and *f*_Other_ for each sample, as shown in Figure 3a–l.

**Figure 3:**
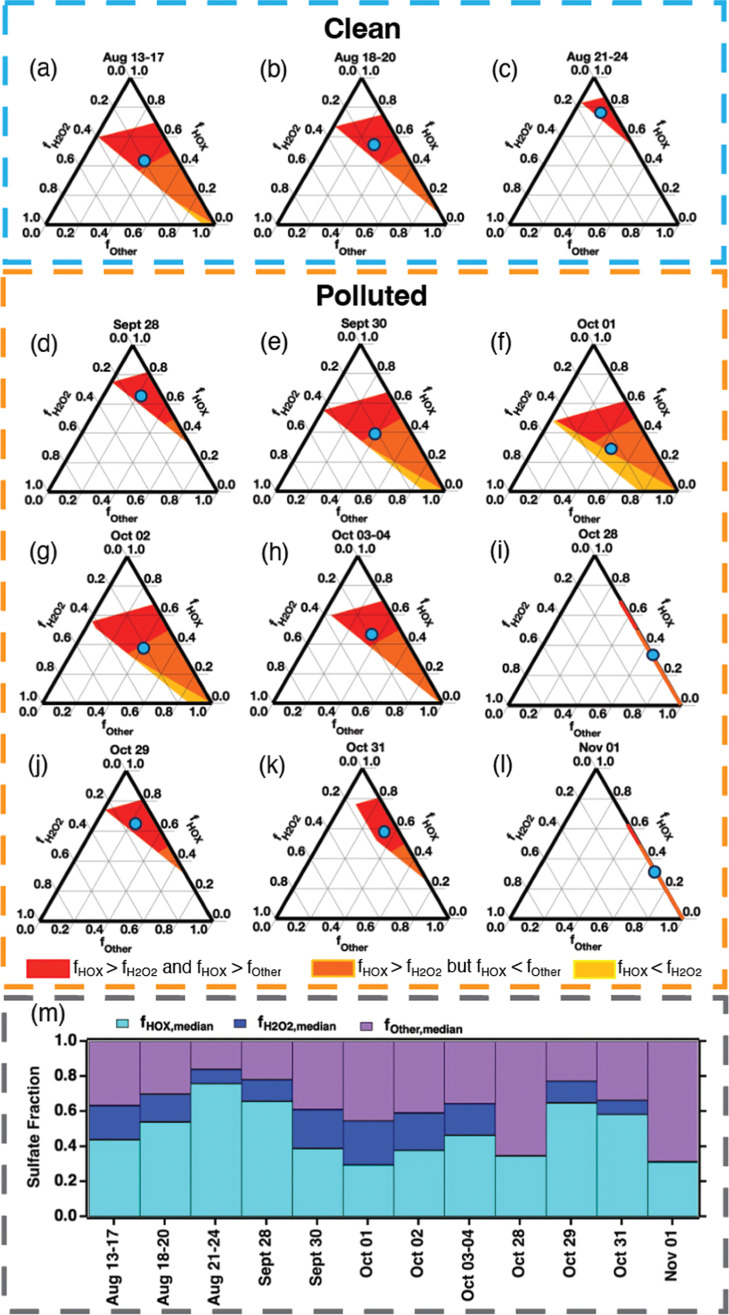
**(a–l)** The possible range of *f*_H2O2_, *f*_HOX_, and *f*_Other_ (= *f*_primary_ + *f*_OH_ + *f*_TMI_ + *f*_O3_) based on observed δ^18^O_nssSO4_ and Δ^17^O_nssSO4_ for each sample shown on a ternary plot. The representative combination scenario with median *f*_HOX_ at median *f*_H2O2_ for each sample is shown in **(m)** and as cyan dot in **(a–l)**.

### 3.2 Sulfate Production via O_3_ and H_2_O_2_ Oxidation Mechanisms

The low Δ^17^O_nssSO4_ and δ^18^O_nssSO4_ in our PM_2.5_ samples indicates very small fraction (<7%) of sulfate produced from O_3_ oxidation within cloud droplets and fine-mode sea salt aerosols. Our data, together with previous observations in the MBL that shows Δ^17^O_nssSO4_ < 1‰ in general,^5,7,8,42^ suggest that O_3_ oxidation is often a minor source of fine-mode aerosol sulfate in both remote and polluted MBL. Since O_3_ oxidation mainly occurs at pH values above ∼5, this supports the recent laboratory experiments that fine sea salt aerosols acidify quickly in the MBL due to uptake of acids such as nitric acid (HNO_3_) and hydrochloric acid (HCl) and dissociation of organic acids.^48^ The fine aerosol pH at the Hok Tsui field site calculated from the E-AIM aerosol thermodynamic model is on average 2.2 ± 0.5 during the study period. Note that Yu et al.^49^ recently suggested that high ionic strength in sea salt aerosols would enhance the oxidation rate of S­(IV) by O_3_. But this contradicts the low Δ^17^O_nssSO4_ values of fine aerosols measured in the MBL so far. Heterogeneous oxidation of SO_2_ by O_3_ could be efficient in coarse-mode sea-salt aerosols,^7,8^ but the oxygen isotopes of sulfate in coarse aerosols were not measured in this study. Nevertheless, coarse-mode (2.5–10 μm) aerosol sulfate mass concentration was on average only 6 ± 5% of fine-mode (<2.5 μm) aerosol sulfate concentration throughout the field campaign.

The observed low Δ^17^O_nssSO4_ and δ^18^O_nssSO4_ for each aerosol sample suggest that, at maximum, the fraction of sulfate produced via H_2_O_2_ oxidation (*f*_H2O2,max_) could only reach 16–39% (on average 29%) for clean air masses from South China Sea and 0–50% (on average 27%) for polluted air masses from the southeast coast of China (Figure 3a–l). This contrasts with the traditional view that H_2_O_2_ is the main oxidant for sulfate production in the MBL.^4,10,50^ A recent box modeling study investigating sulfate formation in the mixed urban and maritime coastal environment also simulates relatively low fraction (30%) of sulfate produced from H_2_O_2_ oxidation.^6^ They calculated HOI as the largest contributor (57%) to sulfate formation.^6^

### 3.3 Sulfate Production via HOX Oxidation Mechanism

In contrast to the high δ^18^O and Δ^17^O of sulfate produced from H_2_O_2_ and O_3_ oxidation, sulfate produced from HOX oxidation possesses the lowest δ^18^O among all pathways as well as a low Δ^17^O (0‰) (Figure 2). The fraction of sulfate produced from HOX oxidation (*f*_HOX_) was compared to that from H_2_O_2_ oxidation (*f*_H2O2_) in Figure 3. In 7 out of 12 samples, *f*_HOX_ was always higher than *f*_H2O2_, suggesting high contributions from HOX to sulfate formation in both clean (Figure 3b,c) and polluted air masses (Figure 3d,i–l). In particular, on Aug. 21–24 with clean air masses from the South China Sea (Figure 1), *f*_H2O2_ ranges from 0–16%, and at minimum 52–83% *f*_HOX_ is needed to explain the oxygen isotopic signatures (Δ^17^O_nssSO4_ = 0.22‰, δ^18^O_nssSO4_ = 3.49‰) (Figure 3c). On Sept. 28 with polluted air masses from the southeast coast of China (Figure 1), *f*_H2O2_ ranges from 0–25%, and at minimum 32–75% *f*_HOX_ is needed to explain the oxygen isotopic signatures (Δ^17^O_nssSO4_ = 0.19‰, δ^18^O_nssSO4_ = 1.75‰) (Figure 3d). In the other 5 out of 12 samples, the probability of *f*_HOX_ > *f*_H2O2_ reaches 96% on Aug. 13–17 (Figure 3a), 89% on Sept. 30 (Figure 3e), 71% on Oct. 01 (Figure 3f), 89% on Oct. 02 (Figure 3g), and 99.7% on Oct. 03–04 (Figure 3h). These results suggest that more sulfate was produced from HOX oxidation than H_2_O_2_ oxidation in the MBL of both the South China Sea and the southeast coast of China during the entire study period.

Based on the probability distribution of the sulfate fraction from different formation pathways, as shown in Figure 3a–l, we estimated a set of representative *f*_H2O2_, *f*_HOX_, and *f*_Other_ for each sample. The mass-independent signal (Δ^17^O) of sulfate is only caused by H_2_O_2_ and O_3_ oxidation (*E*12), so we first use the median values for *f*_H2O2_ (*f*_H2O2,median_) and *f*_O3_ (*f*_O3,median_) for each sample. At *f*_H2O2,median_, we can calculate median *f*_HOX_ (*f*_HOX,median_) and *f*_Other_ (*f*_Other,median_) for each sample, as shown in Figure 3m. The average (±σ) *f*_H2O2,median_, *f*_HOX,median_, and *f*_Other,median_ for all samples are 14 ± 8%, 48 ± 15%, and 38 ± 16%, respectively, during the study period. *f*_O3,median_ is only 2 ± 2% on average, as a part of *f*_Other,median_. *f*_HOX,median_ is on average more than three times greater than *f*_H2O2,median_ (statistically significant, *p* < 0.01) and is not statistically significant different from *f*_Other,median_ (*p* = 0.1), after considering the uncertainties of each sample (Table S6). Thus, our results indicate that HOX oxidation is a more important sulfate formation mechanism than H_2_O_2_ oxidation, though the relative importance of HOX oxidation compared to other pathways (excluding HOX and H_2_O_2_ oxidation) cannot be statistically determined based solely on the oxygen isotopic data.

### 3.4 Other Sulfate Sources

Primary sulfate emitted from fossil fuel combustion as well as secondary sulfate produced from OH and TMI-catalyzed O_2_ oxidation also possess a low Δ^17^O of 0‰, similar to HOX oxidation, but with a higher δ^18^O (Figure 2). These alternative sources could substitute part or sometimes all of the HOX contribution to explain the low Δ^17^O _nssSO4_ and δ^18^O_nssSO4_ (Figure 3a–l). But for some samples even their total contribution is lower than the HOX contribution (Figure 3). In particular, HOX oxidation (*f*_HOX_ = 52–87%) is more important than the sum of primary sulfate, OH oxidation, and TMI-catalyzed O_2_ oxidation (*f*_primary_ + *f*_OH_ + *f*_TMI_ = 0–45%) within clean air masses on Aug. 21–24 (Figure 3c). Similarly, *f*_HOX_ becomes larger than *f*_primary_ + *f*_OH_ + *f*_TMI_ when *f*_H2O2_ is >8% within polluted air masses on Sept. 28 (Figure 3d).

Gas-phase oxidation of SO_2_ by Criegee intermediates was calculated to be a negligible source of sulfate (<0.2%) in our study region.^60^ In addition, Dovrou et al.^11^ reported only a minor increase (<5%) in sulfate production in our study region when considering oxidation of S­(IV) by isoprene hydroxyl hydroperoxides and hydroxymethyl hydroperoxide. Heterogeneous oxidation of SO_2_ by NO_2_ on alkaline aerosols,^12,13^ photosensitized oxidation of S­(IV) by humic-like substances on alkaline aerosols,^14^ and uncatalyzed sulfite oxidation^15^ on alkaline aerosols - which could be significant under polluted haze conditions-are also not considered important sulfate formation mechanisms in this study due to low aerosol pH (average 2.2 ± 0.5). Aerosol pH in South China is typically below 3,^51,61^ which aligns with the low *f*_O3_ values calculated for all samples, as the O_3_ oxidation pathway is only efficient at higher aerosol pH. Furthermore, a recent modeling study found a negligible fraction (<0.1%) of sulfate produced from the oxidation of SO_2_ by NO_2_ on aerosols in our study region.^61^ Following previous studies,^51,62^ interfacial SO_2_ oxidation involving O_2_ on acidic microdroplets under polluted haze conditions^16^ is not considered, as the reported reaction rate appears unreasonable. Oxidation of hydroxymethanesulfonate (HMS) by OH in the aqueous phase is also excluded as a significant sulfate formation mechanism, since the HMS/sulfate ratio is typically <3% in our study region.^52^ Cloud uptake of hydroperoxymethyl thioformate (HPMTF), an oxidation product of DMS, could represent an important sulfate source in the remote MBL.^53^ However, this pathway is expected to be negligible in our study region, where anthropogenic SO_2_ is 1–2 orders of magnitude more abundant than DMS.^1^

### 3.5 Atmospheric Implications

For the first time, we show observational evidence that hypohalous acids could dominate sulfate production in the highly urbanized PRD-HK region, the South China Sea, and southeast coast of China. Such a sulfate source caused by oxidation of SO_2_ by reactive halogens is typically not included in air quality and climate models mainly due to the limited knowledge of HOX abundances. HOCl and HOBr were monitored by an iodide-adduct time-of-flight chemical ionization mass spectrometer (ToF-CIMS) at our site during the study period. The average HOCl mixing ratio was 8 ± 5 ppt in the clean air masses and 55 ± 43 ppt in the polluted air masses (Table 1 and Figure S2), respectively. A comparable level of HOCl (39 ± 19 ppt) was measured at our site during Autumn 2020.^30^ The HOBr mixing ratio was 0.5 ± 0.5 ppt in the clean air masses and 0.7 ± 0.7 ppt in the polluted air masses. Previous modeling studies suggested that HOBr is on the order of 0.1 ppt and HOI is on the order of 1 ppt in the South China Sea and southeast coast of China regions.^55,56^

**Table 1:** Oxidant Concentrations Used to Calculate In-Cloud Sulfate Production in the Clean and Polluted Air Masses

species	clean	polluted	refs
H_2_O_2_ (ppb)	0.2–0.4	0.6–1.2	Guo et al.^67^
O_3_ (ppb)	15 ± 8	55 ± 14	this study
Fe(III) (μM)	0.09 ± 0.02	1.6 ± 0.3	Yang et al.^68^
Mn(II) (μM)	0.09 ± 0.01	0.37 ± 0.04	Yang et al.^68^
HOCl (ppt)	8 ± 5	55 ± 43	this study
HOBr (ppt)	0.5 ± 0.5	0.7 ± 0.7	this study
HOI (ppt)	1–4	1–9	this study

The reaction rate constants of HOCl + SO_3_^2–^ (7.6 × 10^8^ M^–1^ s^–1^), HOCl + HSO_3_^–^ (2.8 × 10^5^ M^–1^ s^–1^), and HOBr + SO_3_^2–^ (5.0 × 10^9^ M^–1^ s^–1^) have been determined in laboratory experiments.^23,24,57^ Based on experimental results of the HOCl + HSO_3_^–^ reaction, Liu and Abbatt^57^ estimated the reaction rate constant of HOBr + HSO_3_^–^ to be 2.6 × 10^7^ M^–1^ s^–1^. The reaction rate constants between HOI and S­(IV) have not been measured but are expected to be higher than those between HOBr/HOCl and S­(IV).^6,58^ The Henry’s law constants for HOCl, HOBr, and HOI are 650 M atm^–1^, 1300 M atm^–1^, and 450 M atm^–1^, respectively.^5,6^ The sulfate production from HOX is highly dependent on cloud pH since HOX reacts much faster with SO_3_^2–^ than HSO_3_^–^ (Figure 4).

**Figure 4:**
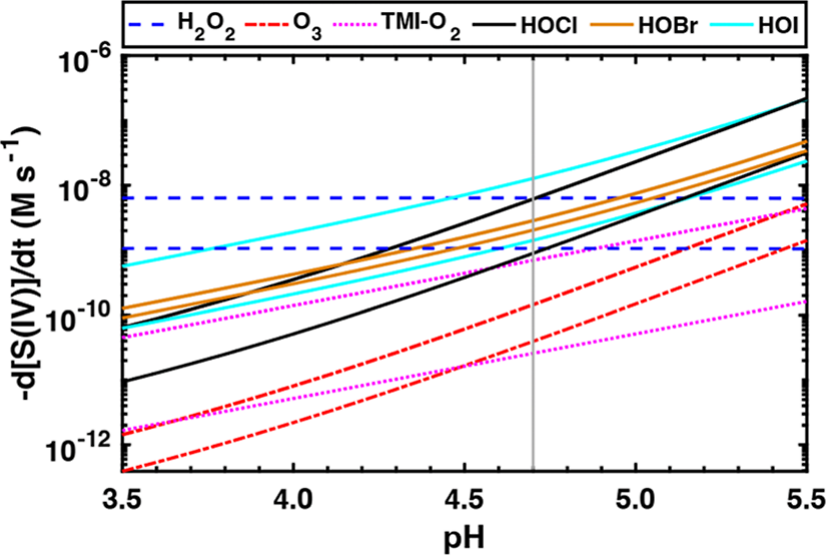
Comparison of sulfate production rate from H_2_O_2_, O_3_, TMI-catalyzed O_2_, HOCl, HOBr, and HOI oxidation at different cloud pH assuming marine boundary layer conditions of [H_2_O_2_] = 0.2–1.2 ppb, [O_3_] = 15–55 ppb, [Fe­(III)] = 0.09–1.6 μM, and [Mn­(II)] = 0.09–0.37 μM, [HOCl] = 8–55 ppt, [HOBr] = 0.5–0.7 ppt and [HOI] = 1–9 ppt. The lower and upper concentrations are associated with the lower and upper lines, referring to the clean and polluted air masses, respectively. TMI is the sum of Fe­(III) and Mn­(II). The cloud pH = 4.7 scenario is highlighted. The cloud liquid water content is assumed to be 0.3 g m^–3^. The reaction rate constants for these sulfate formation mechanisms are shown in Table S3. The Henry’s law constants for H_2_O_2_, O_3_, HOCl, HOBr, and HOI are shown in Table S4.

Assuming a cloud pH range of 3.9–5.4 (Supporting Information Text S1), HOCl of 8–55 ppt and HOBr of 0.5–0.7 ppt could produce sulfate that is comparable to that from H_2_O_2_ oxidation in the MBL (Figure 4). We can further compare different in-cloud sulfate formation mechanisms under typical conditions in this study (cloud pH = 4.7) (Table 1; Supporting Information Text S1–S2): (1) clean air masses: [H_2_O_2_] = 0.2–0.4 ppb, [O_3_] = 15 ± 8 ppb, [Fe­(III)] = 0.09 ± 0.02 μM, [Mn­(II)] = 0.09 ± 0.01 μM, [HOCl] = 8 ± 5 ppt, and [HOBr] = 0.5 ± 0.5 ppt; (2) polluted air masses: [H_2_O_2_] = 0.6–1.2 ppb, [O_3_] = 55 ± 14 ppb, [Fe­(III)] = 1.6 ± 0.3 μM, [Mn­(II)] = 0.37 ± 0.04 μM, [HOCl] = 55 ± 43 ppt, and [HOBr] = 0.7 ± 0.7 ppt. From Figure 3m, the oxygen isotopic signatures of sulfate show that the *f*_HOX,median_/*f*_H2O2,median_ ratio is on average 4.0 in clean air masses and 3.4 in polluted air masses. To reproduce the *f*_HOX,median_/*f*_H2O2,median_ ratios, HOI mixing ratios of 1–4 ppt in clean air masses and 1–9 ppt in polluted air masses are required. Higher HOI in polluted air masses could be due to higher O_3_ to react with I radicals to form IO, which then reacts with HO_2_ to yield HOI, and also higher O_3_ to react with iodide at the ocean surface to produce HOI.^70^ These HOI mixing ratios are on the same order of magnitude as those simulated in a chemical transport model.^56^ In the clean air masses, HOI (48%) dominates sulfate formation via the hypohalous acids pathway, followed by HOBr (36%) and HOCl (16%). In comparison, in the polluted air masses, HOCl (42%) is the biggest contributor of sulfate formation via the hypohalous acids pathway, followed by HOI (38%) and HOBr (20%). The larger HOCl contribution in polluted air masses is due to the much higher HOCl levels (55 ± 43 ppt) compared with clean air masses (8 ± 5 ppt). A recent study showed that HOCl increases with PM_2.5_ nitrate and iron at a coastal city in southeast China during autumn,^71^ implying that aerosol photochemistry involving nitrate and iron releases Cl radicals.^29,72^ These Cl radicals react with O_3_ to form ClO, which then reacts with HO_2_ to produce HOCl. Consistent with this, polluted air masses had higher PM_2.5_ nitrate (1.5 ± 0.6 versus 0.6 ± 0.3 μg m^–3^), PM_2.5_ iron (0.0044 ± 0.0008 versus 0.0012 ± 0.0007 μg m^–3^), and O_3_ (55 ± 14 versus 15 ± 8 ppb) than clean air masses in this study.

In summary, we measured δ^18^O and Δ^17^O of sulfate for aerosol samples collected at coastal Hong Kong and used both to constrain sulfate formation mechanisms. The low sulfate δ^18^O and Δ^17^O observed suggest low contribution of O_3_ and H_2_O_2_ to sulfate production in both clean MBL in the South China Sea and polluted MBL in the southeast coast of China. Instead, HOX oxidation of SO_2_ represented the largest sulfate source. This challenges the traditional view that sulfate is mainly produced from H_2_O_2_, O_3_, TMI-catalyzed O_2_, and OH oxidation in the MBL. Field measurements of HOX and other reactive halogen species, especially in the cloud layer, are essential to assess the importance of this sulfate production mechanism. Furthermore, laboratory experiments are required to determine the reaction rate constants of HOI + S­(IV). The impacts of the HOX oxidation mechanism on the abundances of sulfate aerosols, reactive halogens, and other chemical species in the MBL need to be addressed in the future. In particular, the HOBr + S­(IV) reaction has been identified as a significant sink for reactive bromine in the marine troposphere, leading to decreased BrO and increased O_3_.^55^ The influences of HOCl + S­(IV) on the global reactive chlorine budget - especially on Cl radicals that rapidly oxidize methane (CH_4_) - and the influences of HOI + S­(IV) on the reactive iodine budget - especially on I radicals that reacts with O_3_ - remain poorly constrained. Since reactive chlorine, bromine, and iodine species are strongly coupled, the HOX + S­(IV) reactions can disrupt the entire atmospheric reactive halogen cycle, alter O_3_ and consequently OH levels, and have complex effects on CH_4_ and DMS.^73^

## Supplementary Material



## Notes

Cloud pH estimation (Text S1). Typical concentrations of oxidants for sulfate production (Text S2). Locations of cloudwater and rainwater pH measurement sites (Figure S1). Time series of HOCl mixing ratios (Figure S2). Isotopic signals of HOCl from CIMS (Figure S3). The correlation between δ^18^O of S­(IV) and δ^18^O of the solvent water (Figure S4). Δ^17^O and δ^18^O of sulfate produced via different pathways (Table S1). Average δ^18^O and Δ^17^O values for stable isotope reference materials (Table S2). Reaction rate constants of different aqueous-phase sulfate production mechanisms (Table S3). Henry’s law constants of different oxidants (Table S4). Observational data for each sample (Table S5). The median *f*_H2O2_, *f*_HOX_, and *f*_Other_ values and uncertainties for each sample (Table S6) (PDF)
