# Supplementary material for: Isotopic Evidence Suggests a High Contribution of Hypohalous Acids to Sulfate Formation in the Coastal Marine Boundary Layer
Source: Environ Sci Technol. 2026 Apr 2;60(15):11553–63. doi: 10.1021/acs.est.5c14392 (PMC13104030; doi:10.1021/acs.est.5c14392)
Supplement: Supplementary file 1 [file es5c14392_si_001.pdf]

Isotopic evidence suggests a high contribution of hypohalous acids to sulfate formation in the coastal marine boundary layer

Qianjie Chen[1,], Allison R. Moon[2], Becky Alexander[2], Andrew Schauer[3], Men Xia[1,4], Yifan Jiang[1], Zhouxing Zou[1], and Tao Wang[1,*]*

[1] Department of Civil and Environmental Engineering, The Hong Kong Polytechnic University, Hong Kong SAR 999077, China

[2] Department of Atmospheric and Climate Science, University of Washington, Seattle, Washington 98195, United States

[3] Department of Earth and Space Sciences, University of Washington, Seattle, Washington 98195, United States

[4] Nanjing-Helsinki Institute in Atmospheric and Earth System Sciences, Nanjing University, Suzhou 215000, China

**Correspondence to*: Qianjie Chen (qianjie.chen@polyu.edu.hk) and Tao Wang (tao.wang@polyu.edu.hk)

Number of Pages: 18

Number of Figures: 4

Number of Tables: 6

Text S1. Cloud pH estimation

Only sparse cloud pH measurements are available for Hong Kong and nearby regions (Figure S1). The cloud pH was measured to be 3.9 on average (range 3.0-5.9) at Mt. Tai Mo Shan in Hong Kong located ~25 km northwest of our site during October-November 2016,[1] 4.2 on average (range 4.1-4.4) at Mt. Tianjing in Shaoguan located ~320 km northwest in May 2018,[2] and 3.9 at median level (range 3.4-4.5) at Mt. Lulin in Taiwan located ~700 km northeast during April-May 2011.[3]

According to the Acid Deposition Monitoring Network in East Asia (EANET, https://www.eanet.asia/), during our study period (August-November 2021), rain pH was measured at two sites in Zhuhai, a nearby city ~80 km west of our site, and at two sites in Xiamen on the southeast coast of China (Figure S1), with an average of 5.4±0.3. The rain pH is higher than the cloud pH, likely due to differences in liquid water content, riming, scavenging of aerosols and gases along the precipitation path, and oxidation chemistry within raindrops.[4] It should be noted that there is heterogeneity in both cloud droplet size and pH, and the use of bulk cloud pH typically underestimates sulfate production due to an underestimation of SO_3^{2-}.[5,6] Therefore, in this study, we assume a cloud pH of 4.7 (=3.9/2+5.4/2) and show 3.9 and 5.4 as lower and upper limits, respectively, for calculating the sulfate production rate. This range is consistent with model simulations for southeast coast of China.[4,7]

Text S2. Typical concentrations of oxidants for sulfate production

The H_2O_2 concentration ($[H_2O_2]$) at our site was previously measured to be 0.2-0.4 ppb within clean air masses and three times higher (0.6-1.2 ppb) within polluted air masses in August 2011.[8] $[H_2O_2]$ levels ranging from 0.1 to 1.3 ppb has been measured in the Pearl River Delta – Hong Kong region and modeled in the marine boundary layer (MBL).[9-11] The O_3 concentration ($[O_3]$) at our site, measured using an O_3 analyzer (Model 49i, Thermo Scientific), was on average 15±8 ppb during the clean period (August 13-24) and 55±14 ppb during the polluted periods (September 28 – October 8 and October 28 – November 1). Soluble aerosol Fe and Mn concentrations were measured at the Kowloon Tong site, located about 15 km northwest of our site, to be 0.00147±0.00025 μg m^{-3} and 0.00152±0.00020 μg m^{-3}, respectively, in 2021 summer, and 0.0264±0.0042 μg m^{-3} and 0.0061±0.0007 μg m^{-3}, respectively, in 2021 autumn.[12] Assuming a cloud liquid water content of 0.3 g m^{-3},[1,4] and that all soluble Fe and Mn are present as Fe(III) and Mn(II), the [Fe(III)] and [Mn(II)] concentrations within clouds are calculated to be 0.09±0.02 μM and 0.09±0.01 μM, respectively, in 2021 summer, and 1.6±0.3 μM and 0.37±0.04 μM, respectively, in 2021 autumn. These values represent the upper limits of [Fe(III)] and [Mn(II)]. HOCl mixing ratio was measured at our site to be 8±5 ppt during the clean period and 55±43 ppt during the polluted periods (Figure S2), respectively. HOBr mixing ratio (upper limit) was measured to be 0.5±0.5 ppt during the clean period and 0.7±0.7 ppt during the polluted periods, respectively. HOBr on the order of 0.1 ppt and HOI on the order of 1 ppt over South China Sea and southeast coast of China have been reported in previous modelling studies.[13,14]

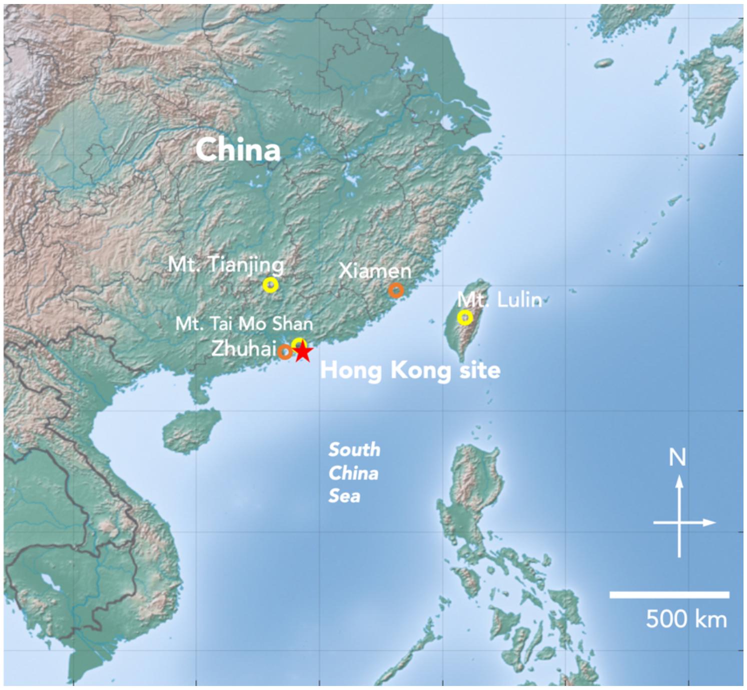

Figure S1. Locations of Zhuhai and Xiamen (orange circles) with rain water pH measured during the study period, as well as Mt. Tai Mo Shan, Mt. Tianjing, and Mt. Lulin (yellow circles) with cloud water pH measured in previous studies.[1-3]

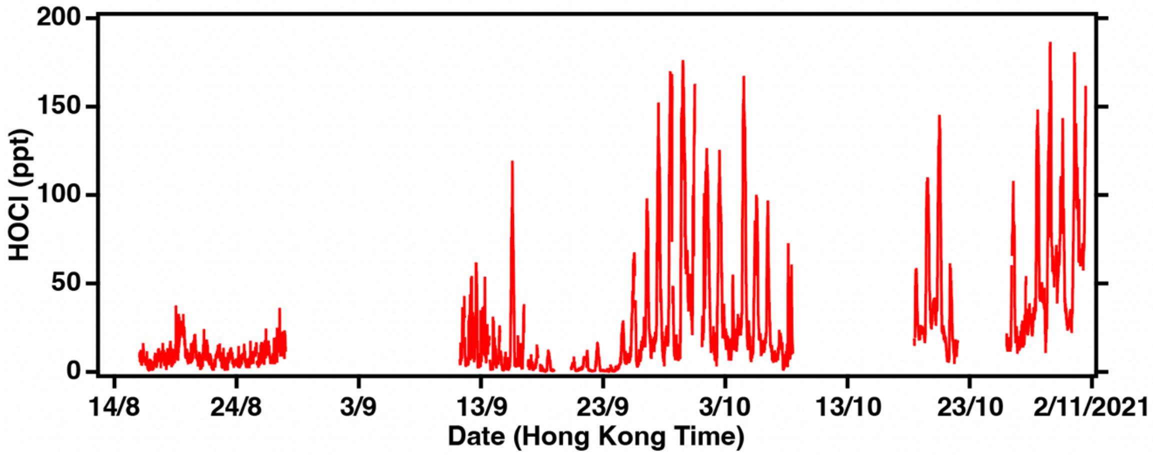

Figure S2. Time series of HOCl mixing ratio at the sampling site at Hok Tsui, Hong Kong during the study period (August-November 2021).

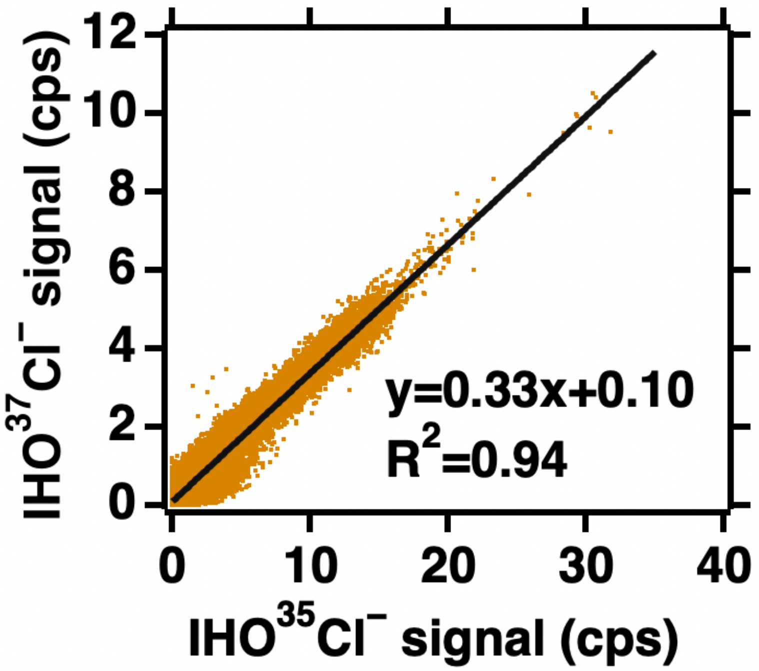

Figure S3. The correlation between $IHO^{37}Cl^-$ and $IHO^{35}Cl^-$ signals showing isotopic ratio used to identify HOCl during the field campaign.

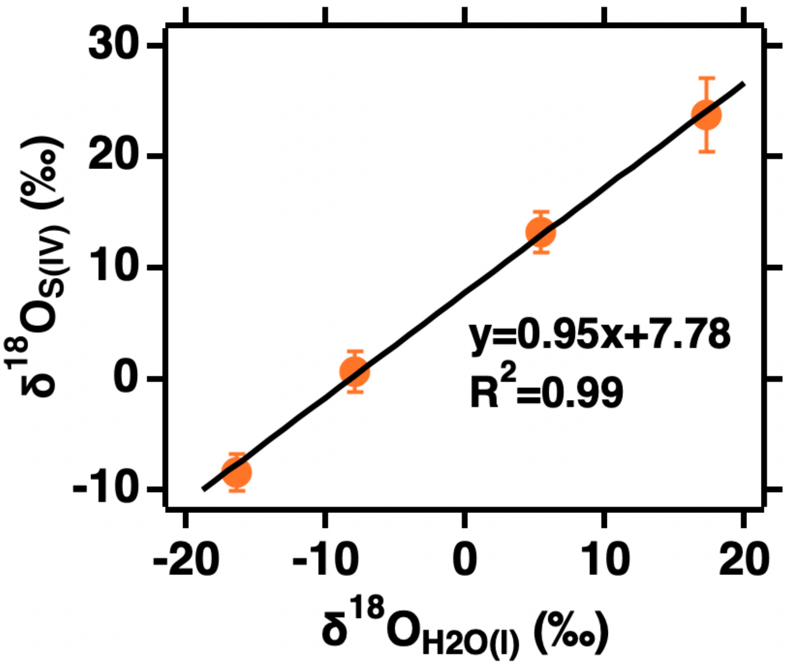

Figure S4. The correlation between $\delta^{18}O$ of S(IV) and $\delta^{18}O$ of the solvent water, using experimental values of S(IV) oxidized by TMI-catalyzed O_2 from Holt et al.[15]

Table S1. $\Delta^{17}O$ and $\delta^{18}O$ of sulfate produced via different pathways. The difference in the $\delta^{18}O$ of product sulfate between Period I and Periods II & III arises from the different $\delta^{18}O$ of rainwater observed in these periods (-6‰ in Period I and -10‰ in Periods II & III). Detailed calculations are provided in Section 2.4.

Sulfate formation pathway	$\Delta^{17}O$ (‰)	$\delta^{18}O$ Period I (‰)	$\delta^{18}O$ Periods II & III (‰)	Reference
SO_2(g)+OH	0	5.9	3.3	16-18
S(IV)+H_2O_2	0.7	18.5	16.8	19,20
S(IV)+O_3	9.8	33.7	31.1	20,21
S(IV)+O_2	0	7.8	5.2	20,22,23
S(IV)+HOX	0	0.1	-3.3	24-26
Primary sulfate	0	23.9	23.9	22,27

Table S2. Average $\delta^{18}O$ and $\Delta^{17}O$ values for stable isotope reference materials measured in both quartz and gold capsules.

Standard	$\delta^{18}O_{quartz}$ (±σ) (‰)	$\delta^{18}O_{gold}$ (±σ) (‰)	$\Delta^{17}O_{quartz}$ (±σ) (‰)	$\Delta^{17}O_{gold}$ (±σ) (‰)
**sulf-alpha**	-6.25 (±1.2)	-7.16 (±0.60)	0.59 (±0.16)	0.86 (±0.15)
**sulf-beta**	-5.41 (±0.90)	-6.78 (±1.1)	1.5 (±0.28)	2.07 (±0.12)
**sulf-epsilon**	-5.52 (±1.40)	-6.71 (±1.2)	5.76 (±0.44)	7.04 (±0.17)
**selenite**	12.65 (±0.66)	12.18 (±0.66)	-0.04 (±0.04)	-0.06 (±0.04)
**IAEA-N-1**	7.83 (±0.68)	5.88 (±0.73)	-0.25 (±0.09)	-0.39 (±0.18)
**s-bravo**	-4.97 (±1.23)	-4.83 (±0.29)	0.44 (±0.07)	0.36 (±0.01)
**s-charlie**	-4.98 (±0.62)	-5.48 (±0.31)	0.67 (±0.09)	0.89 (±0.04)
**s-delta**	-3.95 (±0.51)	-7.39 (±0.24)	2.24 (±0.11)	2.55 (±0.45)
**s-echo**	-4.81 (±0.21)	-5.44 (0.67)	2.38 (±0.02)	3.29 (±0.05)
**s-foxtrot**	-4.01 (±0.77)	-5.89 (±0.94)	6.14 (±0.46)	6.95 (±0.34)
**A220130**	20.41 (±0.68)	21.65	0.07 (±0.1)	0.12

Table S3. Reaction rate constants of different aqueous sulfate production mechanisms.

Aqueous-phase reactions	k_{298} [M^{-1} s^{-1}]	-E_a/R [K]	Reference
$\mathbf{HSO_3^- + H_2O_2 + H^+ \rightarrow SO_4^{2-} + 2H^+ + H_2O}$	$1.5x10^{3(a)}$	-4760	28
$\mathbf{HSO_3^- + O_3 \rightarrow SO_4^{2-} + H^+ + O_2}$	$3.2x10^{5}$	-4830	28
$\mathbf{SO_3^{2-} + O_3 \rightarrow SO_4^{2-} + O_2}$	$1.0x10^{9}$	-4030	28
$\mathbf{S(IV) + O_2 \xrightarrow{Mn(II),Fe(III)} SO_4^{2-}}$	See note[(b)]	/	28
$\mathbf{HSO_3^- + HOCl \rightarrow SO_4^{2-} + 2H^+ + Cl^-}$	2.8×10^{5}	0	29
$\mathbf{SO_3^{2-} + HOCl \rightarrow SO_4^{2-} + H^+ + Cl^-}$	7.6×10^{8}	0	24
$\mathbf{HSO_3^- + HOBr \rightarrow SO_4^{2-} + 2H^+ + Br^-}$	3.2×10^{7}	0	29
$\mathbf{SO_3^{2-} + HOBr \rightarrow SO_4^{2-} + H^+ + Br^-}$	5×10^{9}	0	25
$\mathbf{HSO_3^- + HOI \rightarrow SO_4^{2-} + 2H^+ + I^-}$	3.2×10^{7}	0	11,29,30
$\mathbf{SO_3^{2-} + HOI \rightarrow SO_4^{2-} + H^+ + I^-}$	5×10^{9}	0	11,29,30

[(a)] Rate constant of $\mathbf{HSO_3^- + H_2O_2}$ at pH = 4.7. [(b)] The TMI-catalyzed sulfate production rate is calculate from the following expression: $-\frac{\mathbf{d[SO_4^{2-}]}}{\mathbf{dt}} = \mathbf{750[Mn(II)][S(IV)] + 2600[Fe(III)][S(IV)] + 1.0 \times 10^{10}[Mn(II)][Fe(III)][S(IV)]}$.

Table S4. Henry's law constants of different oxidants.

Oxidant	K_{298} (M atm^{-1})	-Δ***H***°/R (K)	Reference
H_2O_2	7.45x10^{4}	6622	28
O_3	1.13x10^{-2}	2537	28
HOCl	650	/	26
HOBr	1300	/	26
HOI	450	/	11

The Henry's law constant K at temperature T is $K = K_{298} \exp\left[\frac{-\Delta H^{\circ}}{R}\left(\frac{1}{T} - \frac{1}{298.15}\right)\right]$.

Table S5. The measured $\delta^{18}O$ and $\Delta^{17}O$ of $PM_{2.5}$ sulfate, non-sea-salt sulfate fraction of $PM_{2.5}$ sulfate (f_{nssSO4}), calculated $\delta^{18}O_{nssSO4}$ and $\Delta^{17}O_{nssSO4}$, $PM_{2.5}$ mass concentration, $PM_{2.5}$ sulfate mass concentration, and PM_{10} sulfate mass concentration for each sample.

Date	$\delta^{18}O$ (‰)	$\Delta^{17}O$ (‰)	f_{nssSO4}	$\delta^{18}O_{nssSO4}$ (‰)	$\Delta^{17}O_{nssSO4}$ (‰)	$PM_{2.5}$ (μg m^{-3})	$PM_{2.5}$ SO_4^{2-} (μg m^{-3})	PM_{10} SO_4^{2-} (μg m^{-3})
Aug 13-17	7.83	0.33	0.90	7.70	0.37	5.94	1.29	1.41
Aug 18-20	6.41	0.29	0.98	6.35	0.30	6.40	1.80	1.77
Aug 21-24	3.65	0.21	0.97	3.49	0.22	4.82	1.52	1.56
Sept 28	1.87	0.18	0.98	1.75	0.19	22.32	5.62	5.84
Sept 30	5.79	0.33	0.99	5.77	0.33	29.35	8.01	8.19
Oct 01	7.46	0.51	0.99	7.45	0.52	30.51	7.54	7.59
Oct 02	5.72	0.28	0.99	5.68	0.28	19.21	5.34	5.43
Oct 03-04	5.60	0.63	0.95	5.42	0.66	13.26	3.32	3.68
Oct 28	5.23	-0.33	0.95	5.02	-0.35	15.42	4.36	4.94
Oct 29	2.22	0.24	0.97	2.00	0.25	20.51	6.81	7.44
Oct 31	6.05	0.10	0.97	5.95	0.11	24.58	6.67	7.16
Nov 01	7.04	0.00	0.96	6.95	0.00	20.69	5.71	6.27

Table S6. The median f_{H2O2}, f_{HOX}, and f_{Other} values and uncertainties for each sample. The uncertainties refer to half-widths of the 95% confidence intervals.

Date	$f_{H2O2,median}$ (%)	$f_{HOX,median}$ (%)	$f_{Other,median}$ (%)	f_{H2O2} uncertainty (%)	f_{HOX} uncertainty (%)	f_{Other} uncertainty (%)
Aug 13-17*	20	44	36	20	35	40
Aug 18-20	16	54	30	17	33	37
Aug 21-24	8	76	16	11	17	20
Sept 28	12	66	22	14	24	28
Sept 30	22	39	39	23	34	41
Oct 01	25	30	45	25	31	40
Oct 02	21	38	41	22	34	40
Oct 03-04	18	47	36	19	35	40
Oct 28	0	35	65	8	33	34
Oct 29	13	65	23	15	25	29
Oct 31	8	58	34	11	28	30
Nov 01	0	31	69	8	32	33

SI References

1. Li, T.; Wang, Z.; Wang, Y.; Wu, C.; Liang, Y.; Xia, M.; Yu, C.; Yun, H.; Wang, W.; Wang, Y.; Guo, J.; Herrmann, H.; Wang, T. Chemical characteristics of cloud water and the impacts on aerosol properties at a subtropical mountain site in Hong Kong SAR. *Atmos. Chem. Phys.* **2020**, *20* (1), 391–407. DOI: 10.5194/acp-20-391-202
2. Sun, W.; Fu, Y.; Zhang, G.; Yang, Y.; Jiang, F.; Lian, X.; Jiang, B.; Liao, Y.; Bi, X.; Chen, D.; Chen, J.; Wang, X.; Ou, J.; Peng, P.; Sheng, G. Measurement report: Molecular characteristics of cloud water in southern China and insights into aqueous-phase processes from Fourier transform ion cyclotron resonance mass spectrometry. *Atmos. Chem. Phys.* **2021**, *21* (22), 16631–16644. DOI: 10.5194/acp-21-16631-2021
3. Simon, S.; Klemm, O.; El-Madany, T.; Walk, J.; Amelung, K.; Lin, P.-H.; Chang, S.-C.; Lin, N.-H.; Engling, G.; Hsu, S.-C.; Wey, T.-H.; Wang, Y.-N.; Lee, Y.-C. Chemical composition of fog water at four sites in Taiwan. *Aerosol Air Qual. Res.* **2016**, 16, 618–631. DOI: 10.4209/aaqr.2015.03.0154
4. Shah, V.; Jacob, D. J.; Moch, J. M.; Wang, X.; Zhai, S. Global modeling of cloud water acidity, precipitation acidity, and acid inputs to ecosystems. *Atmos. Chem. Phys.* **2020**, *20* (20), 12223–12245. DOI: 10.5194/acp-20-12223-2020
5. Alexander, B.; Allman, D. J.; Amos, H. M.; Fairlie, T. D.; Dachs, J.; Hegg, D. A.; Sletten, R. S. Isotopic constraints on the formation pathways of sulfate aerosol in the marine boundary layer of the subtropical northeast Atlantic Ocean. *J. Geophys. Res.* **2012**, *117*, D06304. DOI:10.1029/2011JD016773
6. Yuen, P.-F.; Hegg, D. A.; Larson, T. V.; Barth, M. C. Paramerization of heterogeneous droplet chemistry for use in bulk cloud models. *J. Appl. Meteorol.* **1996**, *35* (5), 679–689. DOI:

10.1175/1520-0450(1996)035<0679:POHDCF>2.0.CO;2

7. Pye, H. O. T.; Nenes, A.; Alexander, B.; Ault, A. P.; Barth, M. C.; Clegg, S. L.; Collett Jr., J. L.; Fahey, K. M.; Hennigan, C. J.; Herrmann, H.; Kanakidou, M.; Kelly, J. T.; Ku, I.-T.; McNeill, V. F.; Riemer, N.; Schaefer, T.; Shi, G.; Tilgner, A.; Walker, J. T.; Wang, T.; Weber, R.; Xing, J.; Zaveri, R. A.; Zuend, A. The acidity of atmospheric particles and clouds. *Atmos. Chem. Phys.* **2020**, *20* (8), 4809–4888. DOI: 10.5194/acp-20-4809-2020
8. Guo, J.; Tilgner, A.; Yeung, C.; Wang, Z.; Louie, P. K. K.; Luk, C. W. Y.; Xu, Z.; Yuan, C.; Poon, S.; Herrmann, H.; Lee, S.; Lam, K. S.; Wang, T. Atmospheric Peroxides in a polluted subtropical environment: seasonal variation, sources and sinks, and importance of heterogeneous processes. *Environ. Sci. Technol.* **2014**, *48* (3), 1443−1450. DOI: 10.1021/es403229x
9. Duan, L; Jia, S.; Li, X.; Wang, Y.; Zhang, Y.; Fu, S.; Wang, Y.; Ye, C.; Liu, P.; Shi, Z.; Mu, Y.; Recent advancements in observations, sources, and environmental effects of atmospheric hydrogen peroxide (H_2O_2). *Atmos. Environ.* **2025**, *352*, 121230. DOI: 10.1016/j.atmosenv.2025.121230
10. von Glasow, R.; Sander, R.; Bott, A.; Crutzen, P. J. Modeling halogen chemistry in the marine boundary layer, 2. Interactions with sulfur and the cloud-covered MBL. *J. Geophys. Res.* **2002**, *107* (D17), 4323. DOI: 10.1029/2001JD000943
11. Hoffmann, E. H.; Tilgner, A.; Vogelsberg, U.; Wolke, R.; Herrmann, H. Near-explicit multiphase modeling of halogen chemistry in a mixed urban and maritime coastal area. *ACS Earth Space Chem.* **2019**, *3* (11), 2452-2471. DOI: 10.1021/acsearthspacechem.9b00184
12. Yang, J.; Ma, L.; He, X.; Au, W. C.; Miao, Y.; Wang, W.-X.; Nah, T. Measurement report: Abundance and fractional solubilities of aerosol metals in urban Hong Kong – insights into

factors that control aerosol metal dissolution in an urban site in South China. *Atmos. Chem. Phys.* **2023**, *23* (2), 1403–1419. DOI: 10.5194/acp-23-1403-2023

13. Chen, Q.; Schmidt, J. A.; Shah, V.; Jaeglé, L.; Sherwen, T.; Alexander, B. Sulfate production by reactive bromine: Implications for the global sulfur and reactive bromine budgets. *Geophys. Res. Lett.* **2017**, *44* (13), 7069–7078. DOI: 10.1002/2017GL073812
14. Li, Q.; Tham, Y. J.; Fernandez, R. P.; He, X. C.; Cuevas, C. A.; Saiz-Lopez, A. Role of iodine recycling on sea-salt aerosols in the global marine boundary layer. *Geophys. Res. Lett.* **2022**, *49* (6), e2021GL097567. DOI: 10.1029/2021GL097567
15. Holt, B. D.; Kumar, R.; Cunningham, P. T. Oxygen-18 study of the aqueous-phase oxidation of sulphur dioxide. *Atmos. Environ.* **1981**, *15* (4), 557-566. DOI: 10.1016/0004-6981(81)90186-4
16. Dubey, M. K.; Mohrschladt, R.; Donahue, N. M.; Anderson, J. G. Isotope specific kinetics of hydroxyl radical (OH) with water (H_2O): Testing models of reactivity and atmospheric fractionation. *J. Phys. Chem. A.* **1997**, *101* (8), 1494–1500. DOI: 10.1021/jp962332p
17. Lyons, J. R. Transfer of mass-independent fractioation on ozone to other oxygen-containing molecules in the atmosphere. *Geophys. Res. Lett.* **2001**, *28* (17), 3231–3234. DOI: 10.1029/2000GL012791
18. Holt, B. D.; Cunningham, P. T.; Engelkemeir, A. G.; Graczyk, D. G.; Kumar, R. Oxygen-18 study of nonaqueous-phase oxidation of sulphur dioxide. *Atmos. Environ.* **1983**, *17* (3), 625-632. DOI: 10.1016/0004-6981(83)90136-1
19. Savarino, J.; Thiemens, M. H. Analytical procedure to determine both $\delta^{18}O$ and $\delta^{17}O$ of H_2O_2 in natural water and first measurements. *Atmos. Environ.* **1999**, *33* (22), 3683–3690. DOI: 10.1016/S1352-2310(99)00122-3

20. Savarino, J.; Lee, C. C. W.; Thiemens, M. H. Laboratory oxygen isotopic study of sulfur (IV) oxidation: Origin of the mass-independent isotopic anomaly in atmospheric sulfates and sulfate mineral deposits on Earth. *J. Geophys. Res.* **2000**, *105* (D23), 29079–29088. DOI: 10.1029/2000JD900456

21. Vicars, W. C.; Savarino, J. Quantitative constraints on the ^{17}O-excess ($\Delta^{17}O$) signature of surface ozone: Ambient measurements from 50°N to 50°S using the nitrite-coated filter technique. *Geochim. Cosmochim. Acta* **2014**, *135*, 270–287. DOI: 10.1016/j.gca.2014.03.023

22. Barkan, E.; Luz, B. High precision measurements of $^{17}O/^{16}O$ and $^{18}O/^{16}O$ ratios in H_2O. *Rapid Commun. Mass Sp.* **2005**, *19* (24), 3737–3742. DOI: 10.1002/rcm.2250

23. Benson, B. B.; Krause, D., Jr. The concentration and isotopic fractionation of oxygen dissolved in freshwater and seawater in equilibrium with the atmosphere. *Limnol. Oceanogr.* **1984**, *29* (3), 620–632. DOI: 10.4319/lo.1984.29.3.0620

24. Fogelman, K. D.; Walker, D. M.; Margerum, D. W. Non-metal redox kinetics: hypochlorite and hypochlorous acid reactions with sulfite. *Inorg. Chem.* **1989**, *28* (6), 986–993. DOI: 10.1021/ic00305a002

25. Troy, R. C.; Margerum, D. W. Non-metal redox kinetics: hypobromite and hypobromous acid reactions with iodide and with sulfite and the hydrolysis of bromosulfate. *Inorg. Chem.* **1991**, *30* (18), 3538–3543. DOI: 10.1021/ic00018a028

26. Chen, Q.; Geng, L.; Schmidt, J. A.; Xie, Z.; Kang, H.; Dachs, J.; Cole-Dai, J.; Schauer, A. J.; Camp, M. G.; Alexander, B. Isotopic constraints on the role of hypohalous acids in sulfate aerosol formation in the remote marine boundary layer. *Atmos. Chem. Phys.* **2016**, *16* (17), 11433–11450. DOI: 10.5194/acp-16-11433-2016

27. Dominguez, G.; Jackson, T.; Brothers, L.; Barnett, B.; Nguyen, B.; Thiemens, M. H. Discovery

and measurement of an isotopically distinct source of sulfate in Earth's atmosphere. *Proc. Natl. Acad. Sci. U.S.A.* **2008**, *105* (35), 12769–12773. DOI: 10.1073/pnas.080525510

28. Chen, Q.; Sherwen, T.; Evans, M.; Alexander, B. DMS oxidation and sulfur aerosol formation in the marine troposphere: a focus on reactive halogen and multiphase chemistry. *Atmos. Chem. Phys.* **2018**, *18* (18), 13617–13637. DOI: 10.5194/acp-18-13617-2018

29. Liu, T.; Abbatt, J. P. D. An experimental assessment of the importance of S(IV) oxidation by hypohalous acids in the marine atmosphere. *Geophys. Res. Lett.* **2020**, *47* (4), e2019GL086465. DOI: 10.1029/2019GL086465

30. Pechtl, S.; Schmitz, G.; von Glasow, R. Modelling iodide – iodate speciation in atmospheric aerosol: Contributions of inorganic and organic iodine chemistry. *Atmos. Chem. Phys.* **2007**, *7* (5), 1381–1393. DOI: 10.5194/acp-7-1381-2007
